# Effect of pravastatin on the survival of patients with advanced gastric cancer

**DOI:** 10.18632/oncotarget.6777

**Published:** 2015-12-28

**Authors:** Luis Bujanda, Araceli Rodríguez-González, Cristina Sarasqueta, Emma Eizaguirre, Elizabeth Hijona, José J.G. Marín, María J. Perugorria, Jesús M. Banales, Angel Cosme

**Affiliations:** ^1^ Department of Gastroenterology, Donostia University Hospital, Biodonostia Health Research Institute, University of the Basque Country (UPV/EHU), IKERBASQUE, AECC, San Sebastián, Spain; ^2^ Department of Surgery, Donostia University Hospital, Biodonostia Health Research Institute, University of the Basque Country (UPV/EHU), San Sebastián, Spain; ^3^ Donostia University Hospital, Biodonostia Health Research Institute, Red de Investigación en Servicios de Salud en Enfermedades Crónicas (REDISSEC), San Sebastián, Spain; ^4^ Experimental Hepatology and Drug Targeting (HEVEFARM), Biomedical Research Institute of Salamanca (IBSAL), University of Salamanca, Salamanca, Spain; ^5^ National Institute for the Study of Liver and Gastrointestinal Diseases (CIBERehd), Instituto de Salud Carlos III, Madrid, Spain

**Keywords:** gastric cancer, pravastatin, overall survival, advanced

## Abstract

**Objectives:**

A fluoropyrimidine plus cisplatin combined with surgery is standard first-line treatment for advanced gastric cancer. We evaluated the effect of pravastatin on overall survival in patients with advanced gastric cancer in a prospective cohort study.

**Methods:**

At the time of surgery, we assigned 60 patients with advanced gastric cancer (stage III or IV) to receive standard first-line treatment (control group) or standard first-line treatment plus pravastatin at a dose of 40 mg once daily (pravastatin group). The minimum follow-up period was 4 years and the maximum of 6 years.

**Results:**

The mean of age was 66 years and the TNM stage was III and IV in 65% and 35% of patients, respectively. There was no significant difference between the two groups (control vs pravastatin) in median overall survival (15 *vs* 14 months; *P* = 0.8). Predictors of survival were the stage (hazard ratio of death stage IV (III stage as reference): 4.4; 95% CI: 2–9.7; *p* < 0.05) and older age (hazard ratio of death ≥ 65 years (< 65 years as reference): 2.8; 95% CI: 1.3–6; *p* < 0.05).

**Conclusions:**

Pravastatin did not improve outcome in patients with advanced gastric cancer.

## INTRODUCTION

Gastric cancer (GC) is a major cause of mortality from cancer in the world, despite declining rates of incidence in many industrialized countries. Overall, 5-year survival from GC is lower than 30% [1]. One reason for the poor prognosis is that the disease is usually quite advanced by the time symptoms develop. Unfortunately, in Western countries, about 75% of patients have cancer that has spread to the perigastric lymph nodes or have distant metastases at the time of diagnosis. In general, GC appears to be fairly resistant to conventional chemotherapy (5-fluorouracil, cisplatin, and irinotecan, among others) [2].

The role of statins in cancer therapy has been reviewed previously elsewhere. A meta-analysis showed a significant 32% reduction in GC risk with statin use [3]. However, statin use did not improve recurrence-free or overall survival after curative resection for stage II or III gastric cancer in a matched case-control study [4]. In this study, only patients who used statins for more than 6 months had more favourable outcomes than non-users [4]. In mice models, atorvastatin has been shown to block MYC phosphorylatin and activation, suppressing tumour initiation and growth through a HMG-CoA reductase-dependent pathway [5]. The antineoplastic effects of statins have also been demonstrated in human GC cell lines through reduced cell division on whole transcriptome microarrays. Lovastain also induced upregulation of cell-cycle inhibitor p21 and suppression of anti-apoptotic surviving and Mcl-1 proteins in these GC cell lines [6]. Pravastatin is a potent inhibitor of 3-hydroxy-3-methylglutaryl-coenzyme A (HMG-CoA) reductase inhibitor that has been shown to increase survival in patients with advanced hepatocellular carcinoma [7]. Published data indicate that statins can sensitize cancer cells to chemical drugs such as cisplatin and 5-fluorouracil [8]. The objective was to assess the effect on survival of adding treatment with pravastatin in a clinical sample of patients with advanced GC.

## RESULTS

We recruited 60 patients, 38 men and 22 women, with a mean age of 66 years (Table 1). The minimum follow-up period for the living patients was 4 years and the maximum of 6 years. Seventeen patients (28%) were in stage IIIA, 22 (37%) in stage IIIB and 21 in stage IV. Overall, 14 (23%) patients were classified as ASA III.

**Table 1:** Characteristics of the patients included

	Pravastatin group 20 patients (%)	Control group 40 patients (%)	*P* value
Sex			
Male	15 (75)	23 (57.5)	0.26
Age, years	65.4 (± 11.7)	66.3 (± 12.8)	0.8
Stage			
IIIA	7 (35%)	10 (25%)	
IIIB	9 (45%)	13 (32.5%)	0.14
IV	4 (20%)	17 (42.5%)	
ASA class			
II	17 (85%)	29 (72.5%)	0.2
III	3 (15%)	11 (27.5%)	
Surgery			
Yes	18 (90%)	33 (82.5%)	0.7
Chemotherapy			
Yes	7 (35%)	26 (65%)	
Palliative	6 (30%)	5 (12.5%)	0.07
No	7 (35%)	9 (22.5%)	
Radiotherapy			
Yes	5 (25%)	22 (55%)	
No	15 (75%)	18 (45%)	0.03

Table 1 summarises the characteristics of each group. In the pravastatin group, a lower percentage of patients were stage IV (20% *vs*. 42.5%) and were treated surgically (90% *vs* 82.5%), while fewer patients were treated with adjuvant chemotherapy and radiotherapy. Overall, patients in the pravastatin group had better prognostic characteristics, although differences were not statistically significant.

**Figure 1:**
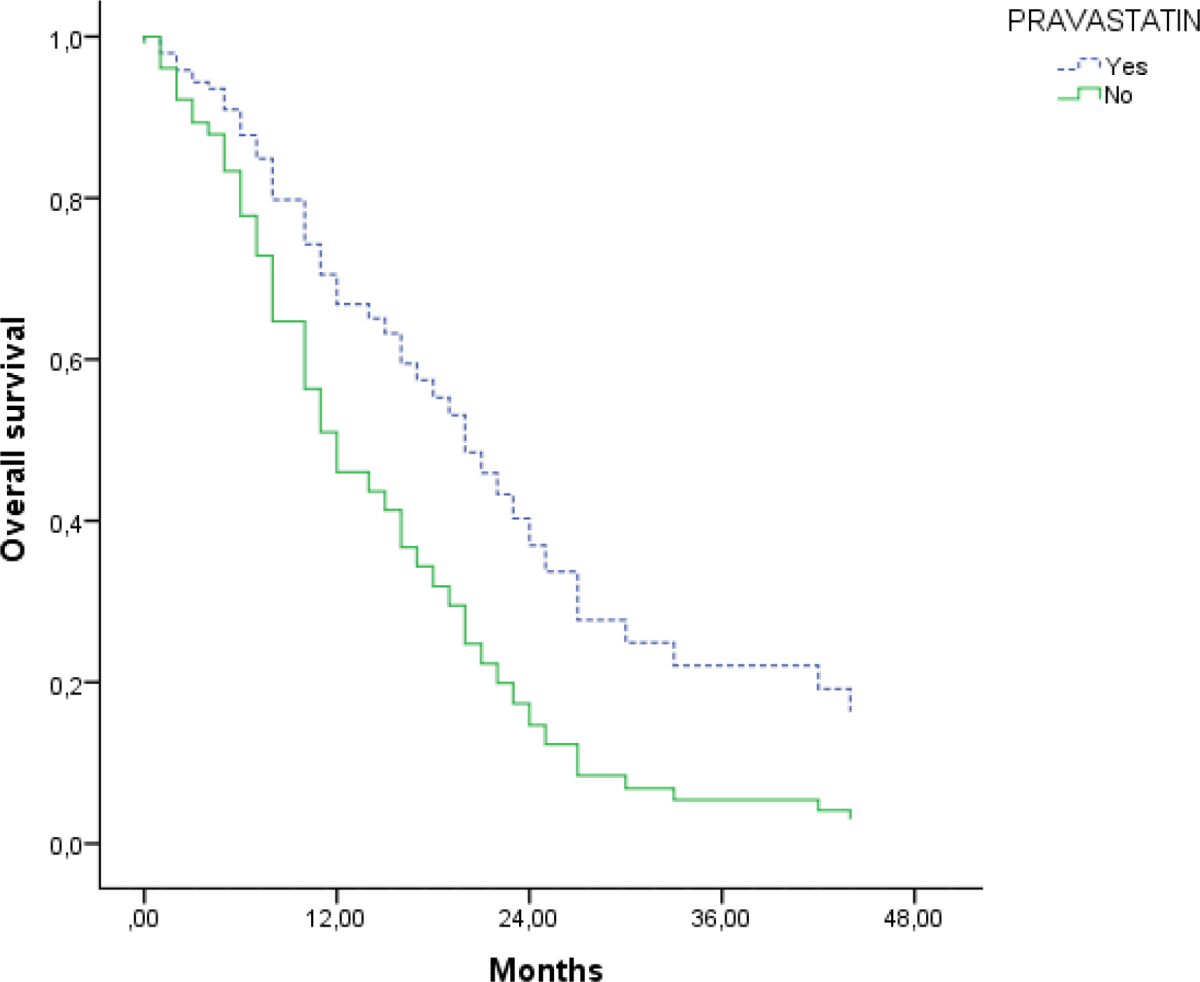
Overall survival adjusted by sex, age, stage, surgery, chemotherapy and radiotherapy

The cumulative probability of survival at 1 year was 55% and 70% in the pravastatin and the control groups respectively, while at 3 years it was 20% and 25%, respectively. The median survival in the pravastatin group was 14 months (95% CI: 5.2–22.7) compared to 15 months (95% CI: 5.7–24.3) in the control group (*p* = 0.8) (Figure 1). After adjusting the analysis by prognostic factors had not significant differences between groups (Table 2).

**Table 2:** Univariate and multivariate Cox regression analysis of prognostic factors for mortality

	Univariate analysis			Multivariate analysis	
	Median overall survival in months (95% confidence interval)	Hazard ratio (95% confidence interval)	*P* value	Hazard ratio (95% confidence interval)	*P* value
Pravastatin					
Yes	14 (5.2–22.8)	Reference		Reference	
No	15 (5.7–24.3)	0.9 (0.5–1.6)	0.8	1.9 (0.8–4.4)	0.1
Sex					
Male	15 (5.9–24.1)	1.5 (0.8–2.9)	0.2	1.8 (0.9–3.6)	
Female	14 (5.7–22.3)	Reference		Reference	0.08
Age, years					
< 65	16.0 (1.9–30.1)	Reference		Reference	
≥ 65	15.0 (6.6–23.3)	1.3 (0.7–2.3)	0.4	2.8 (1.3–6.0)	0.007
Stage					
IIIA	25 (0–50)	Reference			
IIIB	20 (12.0–28.0)	1.5 (0.7–3.2)	0.4	Reference^##^	
IV	6 (4.2–7.8)	4.5 (2.1–9.9)	< 0.0005	4.4 (2.0–9.7)	< 0.0005
ASA class					
2	16 (10.5–21.5)	Reference		—	—
3	6 (0.5–11.5)	1.4 (0.7–2.8)	0.3		
Surgery					
Yes	18 (13.0–23.0)	Reference		Reference	
No	6 (3.0–9.0)	2.1 (1.0–4.6)	0.05	0.8 (0.3–2.2)	0.6
Chemotherapy					
Yes	25 (17.1–32.9)	Reference			
Palliative	8 (4.8–11.2)	3.2 (1.5–6.7)	0.002	Reference*	
No	5 (1.1–8.9)	3.6 (1.7–7.1)	< 0.005	2.8 (0.9–9.4)	0.09
Radiotherapy					
Yes	27 (21.9–32.1)	Reference		Reference	
No	7 (4.6–9.4)	3.1 (1.7–5.5)	0.000	1.9 (0.6–6.1)	0.2

## Reference stage IIIA and IIIB versus stage IV.

* Reference chemotherapy versus palliative and not chemotherapy.

We did not observe any differences in survival as a function of age, sex, or ASA class (Table 2). Surgically-treated patients had a longer survival than other groups (18.8 vs 6.9 months; *p* = 0.05), as did those who received chemotherapy (25.8 months vs 5.3 months; *p* < 0.000) or radiotherapy (27.8 vs 7.1 months; *p* < 0.000). We also found a linear trend in the median survival with stage, median survival being 25.8 months in patients with stage IIIA, 20.8 months in those with stage IIIB, and 6.9 months in those with stage IV disease (*p* < 0.0001). The disease-free survival was 9 months both for stages IIIA and IIIB.

The multivariate analysis showed longer survival in patients under 65 years of age and in patients with stage III compared to those with stage IV disease. Women and the patients who received chemotherapy had a longer survival with a certain trend toward significance statistical (Table 2).

There were no serious adverse events. Two patients had muscle pain in the pravastatin group.

## DISCUSSION

In the present study, we found that pravastatin did not significantly reduce the risk of death or recurrence in patients with advanced gastric cancer. The factors associated with an increase in survival were stage and treatment with surgery or chemotherapy.

Various epidemiological studies have investigated the association between statin use and the risk of gastric cancer, three studies reporting a statistically nonsignificant inverse association [9–11]. However, one study found that ever-use of any statin was associated with significantly lower gastric cancer risk (OR = 0.68) [12]. A systematic review with meta-analysis of eleven studies (seven case-control, one cohort, three post-hoc analysis of 26 clinical trials) evaluating the effect chemopreventive of statins on GC risk showed as the use of statins was associated with a 30% reduction in GC incidence [3]. Other meta-analysis studying the effect of pravastatin therapy on cancer risk did not provide evidence of a link between pravastatin use and cancer risk, with overall rates of cancer of 7.4% in the pravastatin group and 7.0% in the control group [13]. However, the meta-regression analysis indicated that age of study participants significantly modified the association. The risk ratios of cancer associated with pravastatin therapy were 0.99 and 1.22 for ages 60 and 75 respectively.

There are several different mechanisms by which statins might decrease gastric cancer risk or increase survival. One is a reduction in the synthesis of endogenous mevalonate, a compound that is necessary for the biosynthesis of cholesterol and isoprenoid derivates such as farnesyl and geranylgeranyl residues. This depletes mevalonate, a precursor of cholesterol, leading to a reduction in activity of the RAS protein, which is involved in cell differentiation and proliferation [14]. Further, intracellular cholesterol may also provide anticancer effects, as rapidly dividing cancer cells require cholesterol for synthesis of cell membranes [15]. A second mechanism through which statins may interfere with carcinogenesis involves reduced synthesis of mevalonic acid pathway intermediates, such as isoprenoids. Isoprenoids are important in the prenylation and activation of several small GTP-ase cancer signalling pathways, including Ras, Rac and Rho [16]. *In vitro* studies have shown that the inhibition of isoprenoids is one of the mechanisms mediating the effect of statins in cancer [17]. Third, there is also evidence to suggest that statins trigger apoptosis of gastrointestinal cancer cells, inhibit angiogenesis or target mechanisms involved in the metastatic spread of cancer. Fourth, statins inhibited the growth of gastric cancer cells, as well as the volume and weight of tumours in mice. The levels of PCNA was much lower in the group treated with statins [18]. Lastly, recently, it has been observed that statins may also reduce plasma membrane fluidity, particularly in cholesterol-rich rafts, thus interfering with molecular interactions involved in cell signalling emission. A mutated tumour-suppressor protein, p53, has been found to upregulate the mevalonate pathway, an observation that suggests that statins may help revert the malignant phenotype of p53-mutated cancer cells [19]. The levels of ERK1/2, AKT and STAT3 proteins that promote cancer progression were reduced by simvastatin, a finding that correlated with a loss of cell viability and with apoptosis [19].

Studies carried out in humans on the effect of pravastatin on survival for some types of tumours have indicated increased survival in advanced liver cancer, acute myeloid leukaemia and non-serous-papillary epithelial ovarian cancer [20–23]. However, as in our study, other authors have found that pravastatin does not increase overall survival in gastric cancer. Konings et al. [24] assessed the efficacy of pravastatin (40 mg daily) in 30 patients with metastatic gastric carcinoma. The median overall survival was 6 and 8 months in the control and pravastatin group, respectively. These authors concluded that the addition of pravastatin to chemotherapy did not improve outcome in patients with advanced gastric cancer. In contrast to the sample in the aforementioned study, 65% of our patients were in stage III. Thereby, retrospective studies have also found that patients who underwent radical gastrectomy for stage II and III gastric cancer and used statins for less than 6 months did not have more favourable outcomes than non-users [4]. Only in patients with statin use of more than 6 months was this drug associated with increased survival. In that study, the type of statins was unspecified (various statins having been prescribed) and the disease was less advanced (stage II or III). The authors suggest that long-term statin use has chemopreventive effects. Based on these data we think that isn't necessary to include other clinical trials with pravastatin (40 mg once daily) as a treatment associated in patients with advanced GC (stages III or IV). We don't know the effect of pravastatin (40 mg once daily) to long-term in patients with stage I or II, and also, if higher doses (80 mg or 120 mg daily) will decrease mortality and recurrences in patients with stage III or IV.

The differences between gastric cancer and other tumours might be due to not all tumours responding similarly to statins, and secondly, not all statins having the same effect. For example, it has been hypothesized than only lipophilic statins (simvastatin, atorvastatin, lovastatin, fluvastatin, pitavastatin) can inhibit tumour devlopment, while hydrophilic statins (rosuvastatin and pravastatin) would be expected to promote tumour development [21]. In a recent study, the addition of 40 mg simvastatin to capecitabine-cisplatin chemotherapy in patients with advanced gastric cancer did not increase progression-free survival [25]. Thirdly, it may be that to have an effect a higher dose would have been necessary. The dose of pravastatin used for treating patients with acute myeloid leukaemia was 1280 mg per day leukaemia was 1280 mg per day [22]. Fourthly, some authors [26] suggest that pravastatin may promote the development of cancer by inducing mevalonate synthesis in extrahepatic tissues. Fifth, chemotherapy may interact with the effect of pravastatin.

Among the limitations of our research, we should recognise the small number of patients included, and the non-randomised nature of the study. However, we did not observe any tendency towards a greater survival in the pravastatin group, even though this group had better prognostic characteristics, namely, a lower percentage of patients having stage IV disease (20% *vs* 42.5%)

To summarise, pravastatin does not seem to improve survival in patients with advanced cancer patients.

## MATERIALS AND METHODS

We conducted a non-randomised prospective study in clinical practice comparing the effect of pravastatin on survival in patients with advanced GC. The cancer stage was considered advanced in patients with T4 or N1 or M1 disease according to the fourth edition of TNM classification. We included 60 patients admitted to the Department of Surgery between December 2009 and June 2011. All of these patients underwent surgery, their stage being confirmed following surgical. The last date of follow-up was in November 2015. A total of 20 of the patients received pravastatin at a dose of 40 mg/day indefinitely. All patients received the treatment prescribed (surgery and/or chemotherapy and/or radiotherapy and/or palliative care) based on their stage and clinical situation. The chemotherapy drugs used were cisplatin and 5 fluorouracil.

To participate in the trial, patients had to provide written informed consent, to be older than 18 older, and have histologically confirmed gastric cancer (adenocarcinoma) for the first time and at an advanced stage. Exclusion criteria included patients routinely (more than 3 days a week) taking anti-inflammatory drugs or aspirin; receiving oral anticoagulants, fibrates, cyclosporine or oral contraceptives; having hypersensitivity to pravastatin; pregnant women; lactation; having been diagnosed in the previous 5 years with a tumour or diseases with a prognosis of life less than two years.

All patients received the standard treatment (surgery and/or chemotherapy and/or radiotherapy) for each of their clinical conditions. The experimental group received one tablet of 40 mg of pravastatin orally every 24 hours (at breakfast). Patients were monitored every 3 months for at least 2 years, this including laboratory tests. Every 6 months, an abdominal-pelvic CT scan was performed to assess progression or if there was suspicion of progression or cancer recurrence.

The ASA Classification applied was: I - a normal healthy patient; II - a patient with mild systemic disease; III - a patient with severe systemic disease and IV - a patient with severe systemic disease that is a constant threat to life.

The protocol was approved by an institutional review board.

### Statistical analysis

Continuous and categorical variables are presented as medians with ranges and as frequencies with percentages, respectively. Overall survival was estimated using the Kaplan–Meier method. In univariate analysis, potential factors associated with overall survival were explored using the log-rank test. A Cox proportional hazards regression model that included all baseline variables with a *P* value of 0.05 or less for the between-group comparison was used to perform an adjusted analysis of survival. The results are summarized as hazard ratios, 95% confidence intervals, and *P*-values. All *P* values were two-sided, and *P* < 0.05 was considered statistically significant.
